# Transmural rupture and cartilage ring fracture in a tracheobronchial tuberculosis patient

**DOI:** 10.1002/rcr2.495

**Published:** 2019-11-07

**Authors:** Guihui Wu, Qing Chen, Tao Huang, Yong Liu, Wei He

**Affiliations:** ^1^ Department of Tuberculosis The Public Health Clinical Center of Chengdu Chengdu P.R. China

**Keywords:** Balloon dilation, cartilage fracture, tracheal rupture, tracheal stenosis, tracheobronchial tuberculosis

## Abstract

Tuberculosis (TB) tracheobronchial stenosis is considered as the worst complication of tracheobronchial TB (TBTB). Endobronchial balloon dilation (EBD) is a promising treatment for adult tracheal stenosis; however, it may be complicated by transmural rupture and cartilage ring fracture. We present a 29‐year‐old female with a six‐month history of cough and chest pain, and three weeks of dyspnoea. She was diagnosed with TBTB with active caseous lesions and had an effective response to anti‐TB treatment. Nevertheless, she suffered recurrent tracheobronchial stenosis requiring several bronchoscopic treatments, including EBD. Her eight‐month follow‐up bronchoscopy showed transmural rupture and cartilage ring fracture of the anterior trachea. The patient finally recovered after 18 months of conservative management. Transmural rupture and cartilage ring fracture on the anterior trachea wall without pneumomediastinum or subcutaneous emphysema in TBTB patients may be best treated with a conservative approach.

## Introduction

Tracheobronchial tuberculosis (TBTB) is a form of tuberculosis (TB) involving the tracheobronchial tree. TB tracheobronchial stenosis is considered as the worst complication of TBTB. Recently it has become common practice to use bronchoscopic procedures to reduce tracheobronchial stenosis, such as endobronchial balloon dilation (EBD), for they are less invasive and safer than conventional surgery. EBD is the most common intervention in adult stenosis patients; however, its primary disadvantage is the high rate of restenosis and reintervention. Moreover, it may rarely be complicated by the more severe transmural tracheal rupture and cartilage ring fracture. Here, we reported the first case of anterior tracheal transmural rupture and cartilage ring fracture after interventional treatments for TBTB and the entire endoscopic management.

## Case Report

A 29‐year‐old female presented with a six‐month history of cough and chest pain, and three weeks of dyspnoea. She reported no night sweats, weight loss, or haemoptysis. Her past medical history was unremarkable.

**Figure 1:**
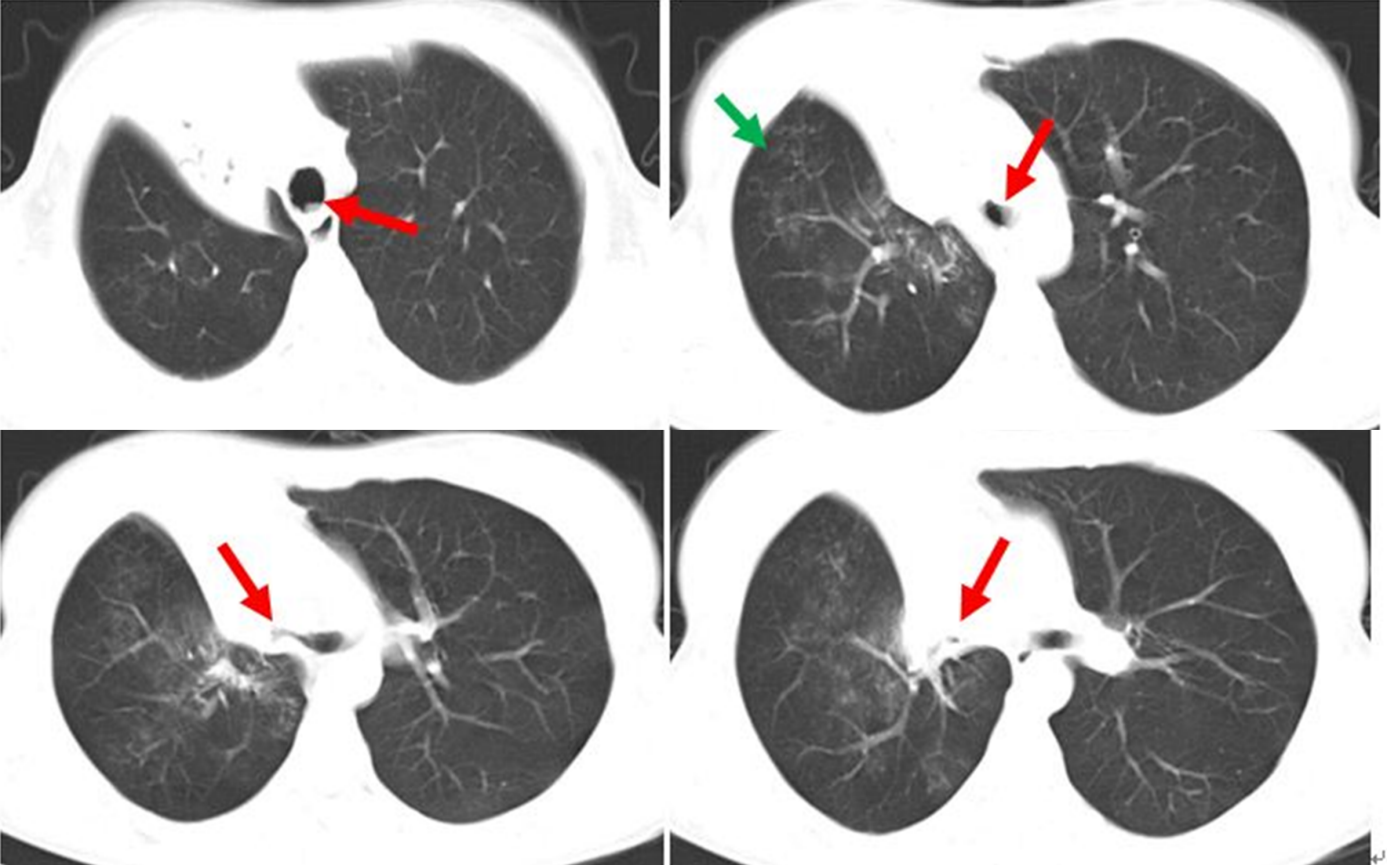
Computed tomography (CT) before treatment. CT scan: Lesions (green arrow) in the pulmonary parenchyma in the right lung; the distal portion of the trachea and the bronchial tree of the right lung were irregular and had varying degrees of stenosis (red arrow).

**Figure 2:**
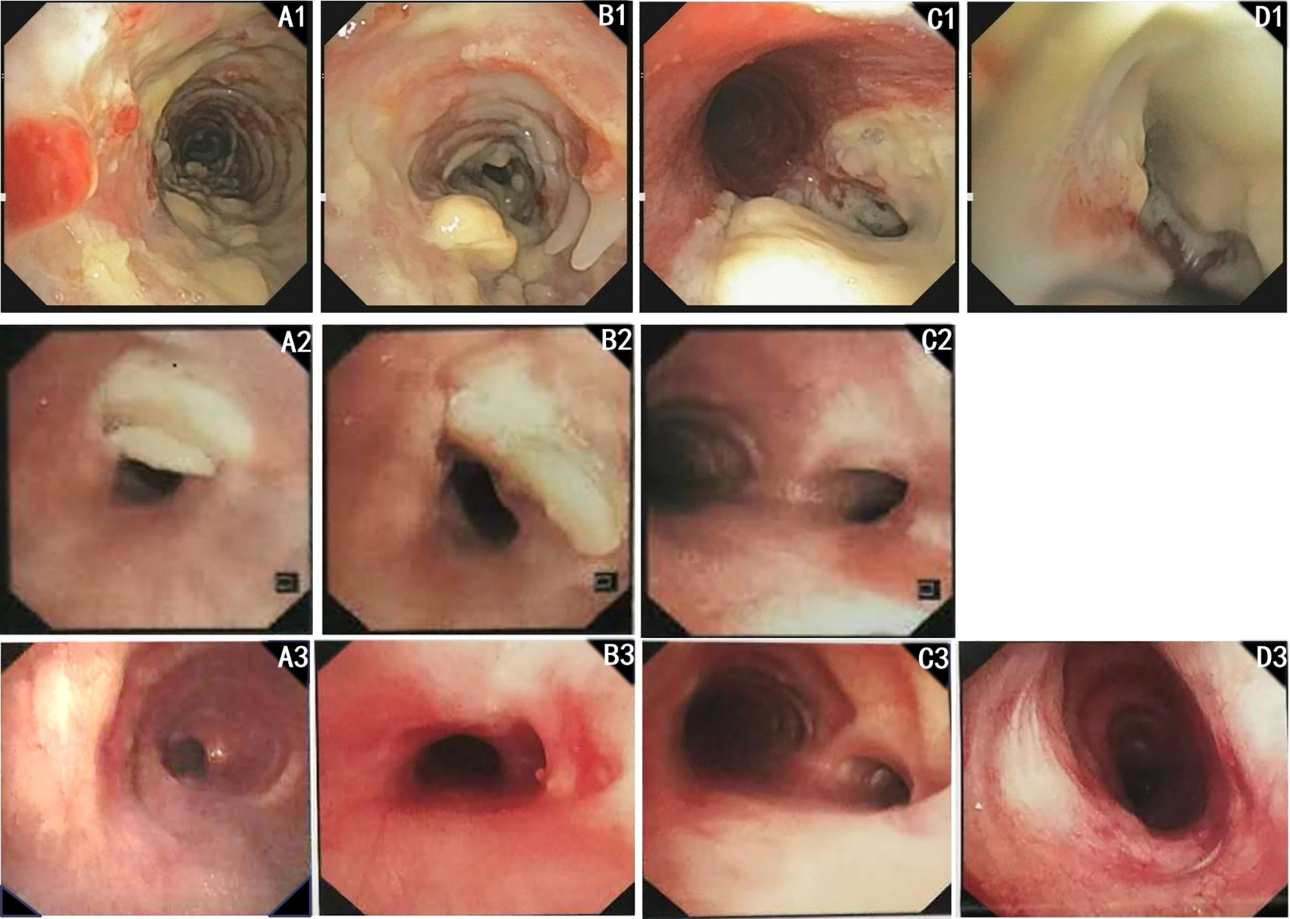
Bronchoscopic examination during the treatment. (A) The upper trachea; (B) the lower trachea; (C) carina; (D) the right main bronchus. A1–D1: Before treatment, all the pictures showed that the lumens of trachea and the right main bronchus were covered with caseous material and the lower trachea was stenosed, 3 cm in length, 51–75% in width; A2–C2: After eight months of treatment, the anterior trachea ruptured and the cartilage ring fractured (7 cm from glottis, 6 cm in length); A3–D3: After one year of treatment, the area of rupture was covered by granulation tissue.

After admission, thoracic computed tomography was evaluated, revealing partial atelectasis of the right lung with parenchymal lesions typical of TB. Furthermore, the lower trachea and the entire visible bronchial tree on the right demonstrated varying degrees of stenosis (Fig. 1). Sputum as well as bronchoalveolar lavage fluid were positive for acid‐fast bacilli on smear microscopy, and cultured drug sensitive *Mycobacterium tuberculosis* (MTB). Xpert MTB/RIF (Cepheid, Sunnydale, California) was positive (rifampicin sensitive) on both respiratory specimens. Bronchoscopy revealed massive caseous material scattered throughout the trachea, causing stenosis in the right main bronchus and right upper lobe bronchus (Fig. 2A1–D1). The diagnosis of TBTB with active caseous lesions was established based on the biopsy result of bronchoscopy.

An 18‐month treatment regimen was constructed, consisting of a 6‐month intensive phase including isoniazid (H) 0.3 g once a day (qd), rifampin (R) 0.45 g qd, ethambutol (E) 0.75 g qd, pyrazinamide (Z) 1.25 g qd, amikacin (Am) 0.4 g qd, and levofloxacin (Lfx) 0.5 g qd, and a 12‐month continuation phase including HREZ, combined with cryotherapy and topical application of HR (H 0.2 g, R 0.15 g) via bronchoscopy.

Two months after treatment, the mucosa of trachea and bronchus improved significantly, but the stenosis of the lower trachea increased. Therefore, EBD and topical application of HR was performed every 7–15 days. However, after two‐month treatment of EBD, the stenosis was recurrent and worsening, with the narrowest portion of the lumen only 3 mm in diameter.

The patient was then transferred to the intensive care unit of the local tertiary hospital, where she underwent electrocautery, argon plasma coagulation, and EBD (15 mm at 2–5 atm) several times. The patient declined stenting. During this period the patient sustained respiratory arrest and required invasive mechanical ventilation. After this the patient developed tracheomalacia, and was subjected to a twice‐weekly regimen of EBD to reduce stenosis and shape the trachea. After eight months of anti‐TB treatment her clinical symptoms had improved significantly; however, unfortunately routine bronchoscopic examination revealed transmural rupture and cartilage ring fracture of the anterior trachea wall (Fig. 2A2–C2, 7 cm from glottis, 6 cm in length), but no haemoptysis, chest pain, or pneumothorax. A small part of tracheal cartilage (11 mm in length, 1 mm in width) was removed (Fig. 2B2) and the rest were able to prevent the airway from collapsing. A conservative management strategy was adopted, as the lesion was over 6 cm long and therefore not amenable to surgery. After 1 year of anti‐TB treatment the area of rupture was covered by granulation tissue, the mucosa had improved significantly and there was no progression of the rupture or ring fracture (Fig. 1A3–D3). Eighteen months after the last EBD, the patient recovered and reported no symptoms related to the tracheal stenosis.

## Discussion

TBTB is one of the main causes of benign airway stenosis in high TB burden country. Bronchoscopic tracheobronchial procedures have become more common at the mainstay of treatment of tracheal stenosis in this population as they are considered safer and less invasive than surgery. However, this case was characterized by the most serious complications of tracheobronchial interventions – transmural tracheal rupture and cartilage ring fracture. While iatrogenic transmural tracheal ruptures (or lacerations) are rare, subclinical lacerations have been reported in up to 50% of tracheobronchial EBD procedures 1. Similarly, Kim et al. found that no transmural tracheobronchial lacerations occurred after EBD 1. In addition, in this cohort, the incidence of airway laceration was significantly higher in females whose strictures were caused by TBTB 1. It is noteworthy that the only other case of transmural rupture and cartilage ring fracture following EBD has been a female diagnosed with TBTB, which may indicate that female and TBTB are two high risks of trachea laceration.

The mechanism of injury to the trachea in EBD is mechanical, but may not be related to the balloon size. A prospective cadaveric study showed that the tracheal cartilage was largely resilient and expansible to balloon dilation (24 mm at 12 atm), with no detectable injury 2.

It has been suggested by Cardillo et al. that the depth of a tracheal rupture is of more clinical importance than the length of the lesion 3. According to a classification system devised by the same author, this patient sustained a level I rupture – without any pneumomediastinum or subcutaneous emphysema, or injury to the oesophagus. These are associated with a better outcome than the more severe forms of rupture. This patient had no distinct change in her symptoms at the time of tracheal rupture and cartilage ring fracture, and recovered with a purely conservative approach. The contribution of topical application of HR or other agents (such as mitomycin C, taxol, or steroids) to the resolution of the stenosis is not addressed here but may need to be explored in prospective trials 4, 5.

### Disclosure Statement

Appropriate written informed consent was obtained for publication of this case report and accompanying images.
